# Prescribing Patterns for the Treatment of Bipolar Disorder in Pregnancy: A Retrospective Cohort Study

**DOI:** 10.3390/jcm14051638

**Published:** 2025-02-28

**Authors:** Nalinoë J. Kernizan, Abigail M. Yancey, Alicia B. Forinash, Niraj R. Chavan, Katherine J. Mathews

**Affiliations:** 1 Department of Pharmacy Practice, St. Louis College of Pharmacy, University of Health Sciences and Pharmacy, St. Louis, MO 63110, USA; nkernizan@vanguardmedgroup.com (N.J.K.); abigail.yancey@uhsp.edu (A.M.Y.); alicia.forinash@uhsp.edu (A.B.F.); 2 SSM Health St. Mary’s Hospital, St. Louis, MO 63132, USA; katherine.mathews@health.slu.edu; 3 Division of Maternal-Fetal Medicine, Saint Louis University School of Medicine, St. Louis, MO 63117, USA; 4 Department of Obstetrics, Gynecology, and Women’s Health, Saint Louis University School of Medicine, St. Louis, MO 63117, USA

**Keywords:** pregnancy/drug effects, antipsychotics (atypical), bipolar disorder, mood stabilizers, antiseizure medications, obstetrics/gynecology

## Abstract

**Background:** Untreated bipolar disorder during pregnancy is associated with poor prenatal care, decreased fetal growth, and an increased risk for postnatal complications, including postpartum psychosis. Although mood stabilizers are first-line therapy, many patients and providers discontinue them in early pregnancy. Antidepressants as monotherapy can increase the risk of mania and rapid cycling, especially in patients with bipolar I, and are not recommended. **Objective**: This study aims to describe prescribing patterns for the pharmacologic management of bipolar disorder in pregnancy. **Methods**: This retrospective cohort study included pregnant patients, ≥14 years old, with a documented diagnosis of bipolar disorder and ≥two clinic visits after 1 January 2014, who delivered by 31 October 2017, within our health system. Eligible patients were identified by the ICD-9 and ICD-10 codes for bipolar disorder and their medication profiles. The primary outcome was to describe bipolar disorder treatment regimens at first visit, during pregnancy, and at delivery. Descriptive statistics were used. **Results**: Of the 214 pregnancies analyzed, 134 (62.6%) used psychiatric medications during pregnancy, with 79/134 (59%) being mood stabilizers. During the initial visit, 61/214 (28.5%) pregnancies were on psychiatric medications, including 30 (49.2%) on mood stabilizers and 16 (26.2%) on antidepressants alone. At delivery, 98/214 (45.8%) pregnancies were on psychiatric medications, with 48/98 (49%) on mood stabilizers and 35/98 (35.7%) on antidepressants without mood stabilizers. Other therapies included benzodiazepines, buspirone, and amphetamines, as monotherapy or combination. **Conclusions**: Despite having documented bipolar disorder, only 30/214 (14%), 79/214 (36.9%), and 48/214 (22.4%) pregnancies were treated with mood stabilizers at the first visit, during pregnancy, and at delivery, respectively. Unfortunately, justification for discontinuation was not documented. The most commonly prescribed mood stabilizer was lurasidone, followed by lamotrigine. Antidepressant monotherapy persisted throughout pregnancy, demonstrating inappropriate disease management.

## 1. Introduction

Bipolar disorder is a medical condition that affects 3.9–6.4% of Americans with an equal distribution between men and women and includes two major subsets based on symptoms and duration (Table 1) [1,2]. Women are typically diagnosed between 18 and 30 years of age, a time when many may become pregnant. A 2022 systematic review and meta-analysis found that 54.9% of women with bipolar disorder had at least one bipolar-spectrum mood episode (featuring depression, mania, hypomania, and/or psychosis) during pregnancy [3]. The risks associated with untreated or inappropriately treated bipolar disorder during pregnancy are significant and include a 25–50% increased risk for postpartum psychosis, mood worsening postpartum, fetal growth restriction, preterm birth, adverse neurodevelopmental outcomes, suicide, and increased rates of filicide [4,5]. Potential causes for untreated and uncontrolled bipolar disorder are assorted but include barriers such as suboptimal education regarding bipolar disorder management by obstetrician/gynecologists, fear of poor clinical outcomes, fear of legal recourse, drug safety concerns by providers and patients, discontinuation of psychiatric medications, and suboptimal drug concentrations due to pharmacokinetic changes experienced during pregnancy (i.e., increased renal clearance and drug metabolism), lack of screening, and misdiagnosis [4,6]. Postpartum relapse rates have also been shown to be higher in patients who were not treated for bipolar disorder during their pregnancy (66%) compared to those who were treated (23%) [7].

In addition to the health complications from untreated bipolar disorder, the economic and societal costs, direct and indirect, are significant for untreated mood disorders. Higher work absenteeism and lower labor force participation can lead to income loss and increased use of public services, leading to increased health care costs. One study evaluated the costs of untreated maternal mental health conditions in Texas’ Medicaid for Pregnant Women. It cost the program USD 962 million over a six-year period [8]. Increasing access to appropriate care has the potential to lead to significant health care cost savings.

Mood stabilizers are the backbone of therapy for bipolar disorder, and preferred agents include lithium (mania, depression, and maintenance), valproic acid (mania, depression, and maintenance), lamotrigine (depression and maintenance), and second-generation antipsychotics (SGAs), also known as “atypical” antipsychotics (mania, depression, and maintenance), according to the 2018 Canadian Network for Mood and Anxiety Treatments (CANMAT). The CANMAT guidelines recognize the inconsistency regarding the medications defined as mood stabilizers, so for this study, mood stabilizers included lithium, lamotrigine, and atypical antipsychotics. [9].

The 2023 American College of Obstetricians and Gynecologists Clinical Practice Guideline (ACOG CPG) states that pregnancy and lactation alone should not be a reason to withhold mental health medications given that without treatment, untreated and inadequately treated bipolar disorder is considered an exposure and carries a sevenfold increased odds of postpartum hospitalization amongst the aforementioned negative outcomes [4]; therefore, making the selection of pharmacotherapy based on efficacy and risk is important in this special population. It is highly recommended that patients continue pharmacotherapy if they have been stable at the time of pregnancy due to the risk of recurrence or exacerbations of mood symptoms, which outweigh the risk of most medication exposures. Due to potential teratogenic risks in pregnancy and the availability of reasonable alternatives, valproic acid should be avoided as a first-line therapy [4]. Lithium in pregnancy is associated with cardiovascular malformations with first trimester use and pregnancy-related pharmacokinetic changes, as well as potential drug interactions and diet changes, which make maintaining levels challenging due to its narrow therapeutic index [4,10,11]. The ACOG has recommended that pregnant patients taking lithium in the first trimester receive a detailed ultrasound examination in the second trimester [4]. Lamotrigine is a preferred option for treatment and its teratogenic potential has been observed to be low when used in pregnancy; however, it requires a slow titration and is not effective for acute mania [9,12].

SGAs such as olanzapine, quetiapine, risperidone, and aripiprazole are generally well tolerated, have varying efficacy on mania and depression symptoms, and have a better adverse effect profile compared to typical antipsychotics [13]. In general, data suggest they are not associated with malformations during pregnancy; however, one large study of 21,751 women using an SGA only found an increased risk of oral clefts with olanzapine and an increased risk in malformations with risperidone and paliperidone [14,15]. SGAs have also been associated with poor neonatal adaptation syndrome (PNAS) [16]. In fact, the National Pregnancy Registry for Psychiatry Medications found the rate for PNAS with SGAs to be like selective serotonin reuptake inhibitors (SSRIs) and serotonin–norepinephrine reuptake inhibitors (SNRIs) [16].

The use of antidepressant therapy, such as SSRIs and SNRIs, can also be seen in patients with bipolar disorder, as many cases of bipolar disorder are initially misdiagnosed as depressive disorder. Antidepressants as monotherapy can increase the risk of mania and rapid cycling, especially in patients with bipolar I, and are not recommended [4]. They can be considered as adjunct therapy to a mood stabilizer to prevent triggering of a manic episode and rapid cycling in patients but should not be used as first-line monotherapy [17].

Lurasidone is an SGA approved for the treatment of bipolar I disorder and was assigned a pregnancy category B label according to the retired Food and Drug Administration (FDA) pregnancy classification system. Because this was the only SGA at the time of this study with the retired pregnancy category B rating, despite only having animal data, it was approved as the preferred SGA during pregnancy for this health system in December 2013 [18]. It also still shared the same FDA warning for PNAS following delivery as other agents in this class; however, at the time of this study, limited publications had reported these symptoms as a drug-associated risk [18,19].

Perinatal psychiatry access programs have been developed nationally and on the state level to support practitioners by offering free education, technical support, consultations, resources, and referrals with the goal of improving the management of perinatal mental health conditions [20]. Implementation of these access programs has been made possible by federal, state, and other funding methods, which has grown the number of programs to twenty-eight state and two national programs. Missouri’s perinatal psychiatry access program (Maternal Health Access Project) became available for enrollment in April 2024 after our data collection was conducted [21].

The aim of this study was to evaluate the prescribing patterns of an interdisciplinary team of physicians, nurse practitioners, and clinical pharmacists treating patients with bipolar disorder in our center during their pregnancy. In light of the CPGs published by the ACOG regarding the management of mental health conditions, we wanted to assess our clinic’s management of bipolar disorder and wish to add additional information regarding mood stabilizer use. Given the risks of antidepressant monotherapy for bipolar disorder, we sought to gather the presence of manic symptoms; however, these data were not documented. The primary outcome was to describe bipolar disorder treatment regimens at first prenatal visit, during pregnancy, and at delivery.

## 2. Materials and Methods

### 2.1. Study Design

Following approval from the Institutional Review Board, this retrospective chart review abstracted and analyzed data from pregnant patients who were at least 14 years of age with a documented diagnosis of bipolar disorder with at least two prenatal care visits at our outpatient prenatal care center between 1 January 2014 and with delivery by 31 October 2017. Patients were identified by two methods: ICD-9 and ICD-10 codes for bipolar disorder diagnosis and medication profiles via electronic medical records. To capture all patients who may be missed due to coding errors, patients were also identified via active medications listed in their medication profile, including antidepressants, anti-anxiolytics, and mood stabilizers, such as atypical antipsychotics and antiseizure medications. Women who delivered outside our health system were excluded, as neonatal outcomes were unavailable.

### 2.2. Data Collection

This study was conducted in a teaching service providing obstetric and gynecological care for patients in both Missouri and Illinois. The clinic, composed of an interprofessional teaching team for a local university obstetrics and gynecology residency and maternal fetal medicine fellowship programs, provided both low- and high-risk obstetric care. Additionally, high-risk patients were referred for transfer of care, co-management of care, or for a consult by their outside physicians at any point during the pregnancy. The care team included physicians, nurse practitioners, clinical pharmacists, dietitians, diabetic educators, nurses, sonographers, genetic counselors, social workers, and financial counselors. The population served included patients with government insurance (Medicaid and Medicare) and those who were uninsured and underinsured. Co-management with specialty physicians including psychiatry is challenging and often involves a long wait time for the patient to be seen; thus, the clinical team often manages these disease states.

Pertinent maternal and neonatal information was collected from the health system electronic health records. This included maternal age at delivery, gestational age at delivery, race, parity, depression status at the first visit at the study clinic based on a validated questionnaire used at the time (Edinburgh Postnatal Depression Screen or Beck’s Depression Score), tobacco use at the first visit, alcohol exposure, and recreational substance use. All medications including dose and frequency were documented at the first visit. Active psychiatric medications during pregnancy and at the time of delivery, prescriber, gestational age at use, dose, and frequency were also collected. Comorbidities prior to pregnancy such as chronic hypertension, hypothyroidism, hyperthyroidism, epilepsy, anxiety, depression, schizophrenia, asthma, diabetes, history of suicide attempt, history of sexual and/or physical abuse, as well as pregnancy complications such as gestational diabetes, pre-eclampsia, and preterm delivery were abstracted from a review of the electronic health records. Descriptive statistics were reported.

## 3. Results

A total of 195 women met the inclusion criteria, which totaled 214 pregnancies and 220 infants. The average age at delivery was 27.4 years, and 99/195 (50.8%) patients identified as non-Hispanic Black. The majority of patients had their initial visit at the clinic during the second trimester of their pregnancy 102/195 (45%), and the most common comorbidities included asthma, 68/214 (31.8%), and chronic hypertension, 41/214 (19.2%) (Table 2). A total of 99/214 (46.3%) pregnancies were complicated by recreational drug use, and of those 71/214 (33.2%) were cannabis alone. The most common psychiatric comorbidities listed were depression, 142/214 (66.4%), and anxiety, 132/214 (61.7%). Nearly half the patients, 89/214 (41.6%), reported some form of sexual and/or physical trauma, and nearly a quarter, 51/214 (23.8%), had a history of a suicide attempt.

At the first visit, 61/214 pregnancies were on at least one psychiatric medication (28.5% of total pregnancies), with the most commonly used agents being mood stabilizers, present in 14% of all pregnancies, and selective serotonin receptor inhibitors/serotonin–norepinephrine reuptake inhibitors (SSRIs/SNRIs) in 11.7% of all pregnancies. Monotherapy use of psychiatric medications was present in 40/61 (65.6%) pregnancies, with 17/40 (42.5%) of those exposed pregnancies on a mood stabilizer alone. The most prescribed mood stabilizers at first visit are listed in Table 3. Antidepressant use without mood stabilizer use was present in 16/214 (7.5%) pregnancies at first visit.

Antepartum use of psychiatric medications at any time in pregnancy was present in 135/214 (63.1%) pregnancies. Mood stabilizers were initiated in 49/214 (22.9%) pregnancies. Additionally, four pregnant patients were on a mood stabilizer at the first visit but had a change made to the initial regimen that included the initiation of an SGA (characterized by either a change to a different mood stabilizer or the addition of another mood stabilizer to the regimen antepartum). This included, for example, a change from levetiracetam to lurasidone and lamotrigine, a change from quetiapine to lurasidone, or a change from lurasidone and oxcarbazepine to lamotrigine, aripiprazole, levetiracetam, and olanzapine. This increased the total number of pregnancies using antepartum mood stabilizer therapy from 30/214 (14%) to 79/214 (36.9%). The reason for the medication changes was not captured. Nearly 40.4% of the mood stabilizer initiations made during pregnancy were in consultation with the clinic pharmacist. Mood stabilizers were recommended by the clinical pharmacist for 20 patients, in whom 18 had no psychiatric medications at first visit and 2 patients were on antidepressant monotherapy. The remaining initiations were conducted by the physician/nurse practitioners at the clinic and/or inpatient/outpatient psychiatry. Buspirone and SSRI/SNRI use increased to a total of 36/214 (16.8%) and 58/214 (27.1%) pregnancies, respectively. Antidepressant monotherapy use was prescribed in 39/214 (18.2%) pregnancies before delivery. Detailed information on the therapeutic agents prescribed can be seen in Table 3.

At the time of delivery, overall psychiatric medication use decreased and was only present in 98/214 (45.8%) pregnancies. Mood stabilizer use decreased by nearly 40% from 79 to 48 pregnancies, SSRI/SNRI use decreased slightly to 49/214 (22.9%) pregnancies, and buspirone to 27/214 (12.6%) pregnancies. Antidepressant use, without mood stabilizers, reduced slightly and was present in 35/214 (16.4%) pregnancies. The reason for discontinuation, including whether the patient self-discontinued or physician discontinued therapy, was not collected due to inconsistent documentation in the chart.

## 4. Discussion

The aim of this study was to describe prescribing patterns for pharmacologic management of bipolar disorder in pregnancy in our clinic. At their first visit in our clinic, 61/214 (28.5%) pregnancies evaluated were exposed to at least one psychotropic therapy, with 30/214 (14.0%) pregnancies exposed to a mood stabilizer and 16/214 (7.5%) pregnancies were on monotherapy antidepressants. During the pregnancy, this increased by 121% (135/214) but decreased by delivery to 98/214 (45.8%), which is still 60.7% higher than at the first visit. Mood stabilizer use increased with recommendations provided by clinic pharmacists in accordance with guideline recommendations.

A 2019 Australian study evaluated prescribing patterns for 535 obstetric patients with severe psychiatric conditions at two antenatal clinics [22]. Of the 218 women with bipolar disorder, most of these patients received 1–3 psychotropic therapies during pregnancy, and only 14.7% received no psychotropic therapy across their pregnancy. Amongst bipolar patients, atypical antipsychotics use across pregnancy was observed in 61% (*n* = 133) of the patients, with quetiapine being the most common. This was followed by 49% (*n* = 107) of women who received an antidepressant and 100 women who received a mood stabilizer or antiseizure medications. Lithium and lamotrigine were the next most used mood stabilizers at 43% each (*n* = 43). Our study revealed a lower overall use of therapy, with a total of 79 patient pregnancies (36.9%) prescribed a mood stabilizer during pregnancy; however, continued use at time of delivery decreased to only 48 pregnancies (22.4%). The higher rate of psychotropic medication use in the Australian study may be related to their study including only patients with severe disorders, whereas our population included all patients [22].

This study found lurasidone to be the most prescribed mood stabilizer antepartum, followed by lamotrigine. However, the high use may be related to lurasidone being the preferred atypical antipsychotic on the formulary for obstetric patients [18]. Only three publications describe the use of lurasidone in pregnancy. The National Pregnancy Registry for Psychiatric Medications including 134 lurasidone exposures did not find an increased risk for malformations [23]. A case report included a 31-year-old female with bipolar II disorder who used lurasidone throughout pregnancy. She delivered a healthy girl at 40 weeks and 5 days (3160 g, 5-min Apgar 9) via c-section. The pregnancy was complicated by preeclampsia, and the infant was in the NICU for 12 h due to labor complications and meconium-stained amniotic fluid. Lurasidone levels decreased during pregnancy [24]. A retrospective cohort study of pregnant women with bipolar disorder treated with mood stabilizers, including lurasidone, did not demonstrate a difference in neonatal or pregnancy outcomes [25]. More pregnancy safety data have been documented with quetiapine, lamotrigine, aripiprazole, olanzapine, risperidone, and ziprasidone [26].

Based on electronic medical chart review, it appears that a portion of discontinuations were patient-directed without input from the medical team. This trend can be concerning, as the discontinuation of mood stabilizers has shown to increase the risk of a mood disorder recurrence. A prospective observational clinical cohort study evaluated the recurrence of a new mood episode in 89 pregnant patients with diagnosed bipolar disorder [27]. The overall risk of at least one recurrence was 71%, with a twofold greater risk in patients that discontinued mood stabilizer therapy. In patients who discontinued therapy, the median time to first episode of a mood recurrence was more than fourfold shorter, and the proportion of ill weeks during pregnancy was five times greater. Of these, 47% of the recurrent episodes occurred in the first trimester. Unfortunately, we were unable to ascertain the time to recurrence based on the clinical documentation in medical records. The discontinuation rate of about 40% in our study is similar to the rates in other literature. Park et al. evaluated any antipsychotic in pregnancy using Medicaid prescription data from 2001 to 2010 from the Centers for Medicare and Medicaid Services [28]. In the 3 months prior to the last menstrual period, a total of 16,608 women (1.1%) filled an atypical antipsychotic prescription. Of these patients, however, only 49.8% filled an additional atypical antipsychotic prescription during their pregnancy, and only 15.4% remained on therapy throughout the entire pregnancy. Depending on the specific atypical agent, between 49.6% and 59.6% of women discontinued therapy during pregnancy. This study is like ours in that there was a high discontinuation rate of therapy during pregnancy; however, there was no information available to determine if therapy discontinuation was under the recommendation of a health care professional or due to self-discontinuation by the patient [28]. A similar Danish study evaluated drug utilization in pregnant women with bipolar disorder between 1997 and 2012 [29]. Consistent with our study, psychotropic drug use also decreased from 54.8% during the 3 months preconception to 36.6% in the third trimester. An additional study performed by Hanley et al. evaluated the pattern of psychotropic medicine in pregnant patients from 2006 to 2011 among patients with private insurance [30]. A total of 343,299 live births were included. Overall, 10.3% (35,303) of women were prescribed a psychotropic medication during pregnancy; however, this number drops to 6.8% when removing women who only filled one prescription during the first trimester. Unlike our study, discontinuation rates did not consistently decrease throughout pregnancy, with 9.9% (*n* = 33,959) of women filling a psychotropic medication during the six months preconception, 7.2% (*n* = 24,776) during the first trimester, 4.6% (*n* = 15,883) during the second trimester, and 5.3% (*n* = 18,161) during the third trimester [30]. However, the initial rate of psychotropic use at first visit in our study was double that found in the Hanley et al. study, which could be one factor leading to a difference in use and discontinuation rates. Hanley et al. also looked at all patients prescribed psychotropic medications regardless of indication, where our study focused on patients with a diagnosis of bipolar disorder.

Despite the evidence that has linked inappropriate antidepressant use to increased episodes of mania, our study revealed that 58/214 (27.1%) patient pregnancies received an antidepressant during the study period; of those, 14.8% of patients were taking an antidepressant without a mood stabilizer at delivery. This rate of antidepressant monotherapy was similar to the 20% seen in the Danish cohort [29]. This finding is of concern, as the use of antidepressants without a concomitant mood stabilizer has shown to increase the risk of mania in patients with bipolar disorder [31]. During pregnancy, 22.6% of patients presenting with a positive depression screen have bipolar disorder if further evaluated [4]. Unfortunately, our study was not designed to capture those that had a manic or other mood episode during pregnancy. We also found that buspirone use significantly increased antepartum and was maintained in 27 (12.6%) pregnancies at the time of delivery; however, nearly 70% of those patients had a comorbidity of anxiety. Benzodiazepine rates did not significantly differ between the first visit and delivery.

This study, while informative, had several limitations. This study was retrospective, and hence subject to inconsistencies in clinical documentation and contingent upon the thoroughness of chart abstraction—as would be the case with any retrospective chart review-based study. Documentation also did not consistently indicate reasons for medication discontinuation during pregnancy nor type of bipolar disorder. This study did not assess for the presence or control of manic symptoms, and documentation showed that providers were often only investigating for the presence of suicidal or homicidal ideation and/or overall mood. This review did not assess medication procurement from each patient’s pharmacy and adherence, since this was beyond the scope of the research aims. We are unable to correlate antidepressant monotherapy use to manic episodes, as these data were not captured due to lack of documentation. Due to the retrospective design of our study, we were unable to request that health care team members document information to more clearly describe the impact of the prescribing changes on mood control. We were able to collect information as part of the documentation for the usual care of our obstetric patients. Multiple confounding variables such as maternal comorbid conditions, other prescription medication prescribed during pregnancy, and recreational drug use by the mother could have affected our findings. Furthermore, we did not undertake a specific cost effective–cost benefit analyses comparing the impact of pharmacotherapy for the treatment of perinatal bipolar disorder, and hence we are unable to comment on considerations pertaining to costs of care. As such, we did not collect information pertaining to economic variables and therefore cannot present a discussion on the economic impact of associated comorbidities in the context of untreated versus treated bipolar disorder. Similarly, we are unable to comment on the impact of the duration of medication treatment on the disease course of each patient. Lastly, this study relied heavily on ICD−9/10 codes for case identification and on clinical documentation for evaluation of the clinical management, and these processes are fraught with inherent limitations that were beyond our control and could have potentially played a role in shaping the results we present.

Despite these limitations, this study contributes to the literature of pharmacological treatment of bipolar disorder throughout pregnancy and captures prescribing patterns at various times rather than a single time point in care. This study provides a real-world view of the use of psychiatric medications during pregnancies in patients diagnosed with bipolar disorder. Given the challenges of our population receiving psychiatric care, our data capture the prescribing patterns from the obstetrics perspective of treating an underserved population. Our study adds to the burgeoning use of lurasidone in the obstetric population, setting the stage for future research evaluating the efficacy of therapy, maternal and neonatal outcomes, and compliance. This study also demonstrates that with the involvement of clinical pharmacists in a multidisciplinary clinical care team, an increase in the prescribing of guideline-recommended therapy is seen and that rates of inappropriate treatment with antidepressant monotherapy are decreased. These findings call for greater emphasis in the development and implementation of specific care pathways for managing perinatal mental health conditions by a team of specialists, including but not limited to physicians with training in obstetrics and gynecology, maternal fetal medicine, reproductive psychiatry, clinical pharmacists, behavioral health therapists, peer support specialists, and perinatal social workers.

The recent call for the increased involvement of obstetrician/gynecologists in the management of maternal mental health conditions was described in recent ACOG guidelines. The findings of this study highlight the need for further training in the screening and proper management of various psychiatric conditions, particularly bipolar disorder, and the value of interdisciplinary care in the management of obstetric patients with personnel such as psychiatrists, social workers, psychologists, nurses, and pharmacists. Traditionally, obstetricians/gynecologists have not felt comfortable prescribing psychiatric medications, but with the prevalence of mental health conditions they will need to appropriately initiate and maintain patients on therapy. The expanded presence of perinatal psychiatry access programs, which was not available in Missouri until April 2024, coupled with collaboration with clinical pharmacists, presents an opportunity to bolster obstetrician/gynecology resident confidence in prescribing medications for various psychiatric conditions, enhance understanding around the teratogenic risks of psychiatric conditions, improve patient medication compliance, and resolve drug therapy problems in a high-risk, vulnerable, special population.

## 5. Conclusions

The aim of our study was to provide insight into the most prescribed therapies in pregnant patients with bipolar disorder in our clinic. At the first visit, less than one-third of patients were on therapy of any kind. During pregnancy, the prescribing of mood stabilizers increased to almost 40% of patients; however, use decreased to 25% of patients at time of delivery. The most commonly used mood stabilizer was lurasidone, followed by lamotrigine. Monotherapy with antidepressant use persisted throughout pregnancy. There is room for improvement in screening for mania, documenting psychiatric medication use, and ascertaining reasons for medication discontinuation. Future considerations will include analyzing reasons for medication discontinuation, rates of postpartum psychosis, and maternal and neonatal outcomes from exposure to different psychiatric therapies.

## Figures and Tables

**Table 1:** Bipolar disorder classification and symptoms [2].

	Symptoms
Bipolar I	Manic (≥7 days or 1 hospitalization) or mixed episodes and depressive episodes Three or more mania symptoms: increased energy, elevated mood, racing thoughts, decreased need for sleep, grandiosity, excessive talkativeness, reckless behavior, and distractibility
Bipolar II	Hypomanic (≥4 days), mixed episodes, and depressive episodes (≥1 and more common)

**Table 2:** Baseline Demographics (*n* = 214 pregnancies).

Number of Included Women/Pregnancies	195/214	
Number of Infants (*n*)	220	
Average Age at Delivery (years)	27.4	
Number of Minors (<18 years old)	3	
Average Gestational Age at Delivery	37.49 weeks	
Median Parity (Interquartile Range)	1 (0–3)	
Trimester at 1st Visit (*n*; %)	1st—73 (34.1%) 2nd—102 (47.6%) 3rd—39 (18.2%)	
Non-Hispanic Black Race (*n*; %)	99 (50.8%)	
White Race (*n*; %)	92 (47.2%)	
Singleton Births (*n*; %)	208 (97.2%)	
Smokers at 1st Visit (*n*; %)	140 (65.4%)	
Smokers at Delivery (*n*; %)	106 (49.5%)	
Alcohol Exposure During Pregnancy (*n*; %)	13 (6.07%)	
**Recreational Drug Use During Pregnancy (*n*; %)**	99 (46.3%)	
Cannabis (*n*; %)	71 (71.1%) at first visit	28 (39.4%) at delivery
Heroin (*n*; %)	31 (31%) at first visit	3 (9.7%) at delivery
Cocaine (*n*; %)	15 (15.2%) at first visit	4 (26.7%) at delivery
Methamphetamines (*n*; %)	12 (12%) at first visit	2 (16.7%) at delivery
Opioids (*n*; %)	12 (12.1%) at first visit	6 (50%) at delivery
**Comorbidities Present in Each Pregnancy**		
Asthma (*n*; %)	68 (31.8%)	
Hypertension (*n*; %)	41 (19.2%)	
Epilepsy (*n*; %)	20 (9.3%)	
Type 2 Diabetes Mellitus (*n*; %)	12 (5.6%)	
Hyperthyroidism (*n*; %)	6 (2.8%)	
Hypothyroidism (*n*; %)	8 (3.7%)	
**Psychiatric Comorbidities**		
Depression (*n*; %)	142 (66.4%)	
Anxiety (*n*; %)	132 (61.7%)	
Sexual and/or Physical Abuse (*n*; %)	89 (41.6%)	
Suicide Attempt (*n*; %)	51 (23.8%)	
Other (Attention Deficit Hyperactivity Disorder, Obsessive Compulsive Disorder, Borderline Personality Disorder) (*n*; %)	29 (13.6%)	
Schizophrenia (*n*; %)	29 (13.6%)	
Post-traumatic Stress Disorder (*n*; %)	18 (8.4%)	

**Table 3:** Psychiatric Medication Use Throughout Pregnancy of the 214 Total Pregnancies (*n* = Number of Pregnancies).

	First Visit (*n*; %)	During Pregnancy (*n*; %)	At Delivery (*n*; %)
Any Psychiatric Medication	61 (28.5%)	135 (63.1%)	98 (45.8%)
SSRI/SNRI	25 (11.7%)	58 (27.1%)	49 (22.9%)
Trazodone	1 (0.5%)	4 (1.9%)	4 (1.9%)
Bupropion	2 (0.9%)	8 (3.7%)	3 (1.4%)
Mood Stabilizers	30 (14%)	79 (36.9%)	48 (22.4%)
Buspirone	6 (2.8%)	36 (16.8%)	27 (12.6%)
Benzodiazepines	13 (6.1%)	19 (8.9%)	14 (6.5%)
Amphetamine	4 (1.9%)	4 (1.9%)	2 (0.9%)
Hydroxyzine	7 (3.3%)	9 (4.2%)	6 (2.8%)
TCA	3 (1.4%)	2 (0.9%)	1 (0.5%)
Mood Stabilizer Use per Pregnancy *			
Lurasidone	8 (3.7%)	31 (14.5%)	18 (8.4%)
Lamotrigine	8 (3.7%)	21 (9.8%)	13 (6.1%)
Aripiprazole	0 (0%)	10 (4.7%)	4 (1.9%)
Quetiapine	6 (2.8%)	9 (4.2%)	4 (1.9%)
Levetiracetam	5 (2.3%)	7 (3.3%)	4 (1.9%)
Gabapentin	3 (1.4%)	6 (2.8%)	3 (1.4%)
Risperidone	1 (0.5%)	1 (0.5%)	2 (0.9%)
Oxcarbazepine	1 (0.5%)	1 (0.5%)	0 (0%)
Olanzapine	1 (0.5%)	3 (1.4%)	2 (0.9%)

* Patients may have been on more than 1 mood stabilizer.

## Data Availability

The original contributions presented in this study are included in the article. Further inquiries can be directed to the corresponding author(s).

## Abbreviations

The following abbreviations are used in this manuscript:

CPG	Clinical Practice Guideline
PNAS	Poor neonatal adaptation syndrome
SGA	Second-generation antipsychotics
SNRI	Serotonin–norepinephrine reuptake inhibitors
SSRI	Selective serotonin receptor inhibitors
TCA	Tricyclic antidepressants
